# A Remarkable Age-Related Increase in SIRT1 Protein Expression against Oxidative Stress in Elderly: *SIRT1* Gene Variants and Longevity in Human

**DOI:** 10.1371/journal.pone.0117954

**Published:** 2015-03-18

**Authors:** Ulkan Kilic, Ozlem Gok, Ufuk Erenberk, Mehmet Rusen Dundaroz, Emel Torun, Yasar Kucukardali, Birsen Elibol-Can, Omer Uysal, Tolga Dundar

**Affiliations:** 1 Department of Medical Biology and Regenerative and Restorative Medicine Research Center (REMER), International School of Medicine, Istanbul Medipol University, Istanbul, Turkey; 2 Department of Pediatrics, Faculty of Medicine, Bezmialem Vakif University, Istanbul, Turkey; 3 Department of Internal Medicine, Faculty of Medicine, Yeditepe University, Istanbul, Turkey; 4 Department of Medical Biology, Faculty of Medicine, Bezmialem Vakif University, Istanbul, Turkey; 5 Department of Biostatistics, Faculty of Medicine, Bezmialem Vakif University, Istanbul, Turkey; 6 Department of Neurosurgery, Faculty of Medicine, Bezmialem Vakif University, Istanbul, Turkey; West Virginia University School of Medicine, UNITED STATES

## Abstract

Aging is defined as the accumulation of progressive organ dysfunction. Controlling the rate of aging by clarifying the complex pathways has a significant clinical importance. Nowadays, sirtuins have become famous molecules for slowing aging and decreasing age-related disorders. In the present study, we analyzed the *SIRT1* gene polymorphisms (rs7895833 A>G, rs7069102 C>G and rs2273773 C>T) and its relation with levels of SIRT1, eNOS, PON-1, cholesterol, TAS, TOS, and OSI to demonstrate the association between genetic variation in *SIRT1* and phenotype at different ages in humans. We observed a significant increase in the SIRT1 level in older people and found a significant positive correlation between SIRT1 level and age in the overall studied population. The oldest people carrying AG genotypes for rs7895833 have the highest SIRT1 level suggesting an association between rs7895833 SNP and lifespan longevity. Older people have lower PON-1 levels than those of adults and children which may explain the high levels of SIRT1 protein as a compensatory mechanism for oxidative stress in the elderly. The eNOS protein level was significantly decreased in older people as compared to adults. There was no significant difference in the eNOS level between older people and children. The current study is the first to demonstrate age-related changes in SIRT1 levels in humans and it is important for a much better molecular understanding of the role of the longevity gene *SIRT1* and its protein product in aging. It is also the first study presenting the association between SIRT1 expression in older people and rs7895833 in *SIRT1* gene.

## Introduction

*Sirtuin 1 (SIRT1)*, the longevity gene, is located on chromosome 10q21.3 and consists of 11 exons and 10 introns (PUBMED gene, updated on 30-Nov-2014). SIRT1 protein is known as an NAD^+^-dependent histone deacetylase and acts as a transcription factor and a cofactor in addition to being a target for histone and non-histone proteins [1, 2]. SIRT1 protects cells against oxidative stress, regulates glucose/lipid metabolism, and promotes DNA stability by binding to and deacetylating several substrates [3]. Because of these protective roles against several age-related pathologies, SIRT1 is thought as one of the candidate molecules for promoting healthy aging.

Sirtuins function as slowing aging and decreasing various age-related disorders, including metabolic diseases, cancer, and neurodegenerative conditions [4]. In previous lifespan studies done using yeast, worms and flies as model organisms, it was found that sirtuins are evolutionarily conserved mediators of longevity [5–7]. Also, a previous study performed with SIRT1 knockout mice showed a significantly shorter lifespan compared with wild-type mice [8]. The effect of SIRT1 against age-related diseases for lifespan elongation may occur by increasing stress resistance and protecting cell death. For example, Alcendor et al. [9, 10] reported that the protective effects of SIRT1 are because of its inhibitory effect on p53 acetylation which stimulates cell death mechanisms and its stimulatory effect on cytoprotective- and stress resistance-genes activator FoxO1a which prevents oxidative stress by upregulating catalase activity.

Most of previous researchers have described a key role for SIRT1 in regulating the metabolic response to calorie restriction, a dietary regimen involving reduced 30–40% calorie intake without malnutrition, which resulted in extended lifespan and reduced development of morbidity with aging [11–14]. However, applying the longevity research of calorie restriction to humans is difficult because it is impossible to conduct a randomized, diet-controlled long-term survival study in humans. Therefore, determination of how the level of SIRT1 changes at different ages may give a clue about the relation between SIRT1 level and lifespan elongation in humans. In one of our studies, in the patients with cardiovascular diseases (mean age: 58.99 ± 10.56), we found a positive relation with SIRT1 protein and age [15] whose role in aging is also gaining importance by other researches [10, 16, 17]. However, most of the studies related SIRT1 expressions were performed either on animal models [18–20] or in cell culture of human tissue [21–23]. In literature, there is almost no study showing how SIRT1 level changes in humans from childhood to elderly. Therefore, in the present study, we planned to determine SIRT1 level in three different age groups (children, adults and older people) to investigate how its level changes during aging. Nowadays, the epigenetic modification of genes also becomes a hot subject for gene-environment interactions to provide essential life styles by decreasing the risk of age-related diseases. It is known that individual variation due to epigenetic factors have a considerable interaction with observed phenotypes [24]. Therefore, the aim of this study was to investigate the association between *SIRT1* single nucleotide polymorphisms (rs7895833 A>G in the promoter region, rs7069102 C>G in intron 4 and rs2273773 C>T in exon 5 silent mutation) and levels of SIRT1 and eNOS expression, as well as paraoxonase-1 (PON-1) expression, cholesterol, total antioxidant status (TAS), total oxidant status (TOS) and oxidative stress index (OSI) with age in Turkish population.

## Experimental Methods

### Study groups

The study groups consisted of randomly selected healthy 120 children (57 male/ 63 female) (age: 8.6 ± 0.3; range is 3–16) with BMI of 17.23, 115 adults (103 male/ 12 female) (age: 46.9 ± 0.5; range is 32–55) with BMI of 28.30, and 103 older people (67 male/ 36 female) (age: 73.5 ± 1.0; range is 56–92) with BMI of 26.10. The randomly selected subjects were recruited from people who came to Bezmialem Vakif University Hospital for routine examination. The older people have normal triglyceride, total cholesterol, HDL-cholesterol, LDL-cholesterol, and fasting blood glucose (Table 1).

**Table 1:** Clinical characteristics of the study population.

	**Children (n = 120)**	**Adults (n = 115)**	**Older (n = 103)**
Triglyceride (mg/dl)	77.25 ± 4.42	165.84 ± 12.14	126.26 ± 6.72
Total cholesterol (mg/dl)	151.00 ± 2.67	184.24 ± 4.85	179.90 ± 4.30
HDL cholesterol (mg/dl)	56.47 ± 1.25	43.59 ± 2.17	38.18 ± 1.18
LDL cholesterol (mg/dl)	87.28 ± 2.23	135.68 ± 3.77	106.44 ± 3.70
Fasting blood glucose (mg/dl)	88.86 ± 0.84	118.00 ± 5.89	103.63 ± 2.58

n, number of individuals. The results are shown as mean ± Standart Error of Mean (SEM). HDL, high density lipoprotein; LDL, low density lipoprotein.

This study was approved by the Ethical Committee of Bezmialem Vakif University, Faculty of Medicine (01.02.2012–7/15; 08.08.2012–21/25; 08.04.2013–36/13). Next of kins, adults and older people, after giving written informed consent, completed a written structured questionnaire in order to collect demographic data. The study was conducted in accordance with the ethical principles described by the Declaration of Helsinki.

### Determination of SIRT1 and eNOS protein levels

After 12 h of fasting, blood samples of subjects were taken into tubes (Vacuette, Greiner Labor technic, Germany) and centrifuged for 5 min at 4°C. Then, blood serum and plasma were collected and stored at -20°C. Plasma/serum samples of subjects were analyzed for levels of SIRT1 and eNOS proteins using enzyme-linked immunosorbent assay (ELISA) kits from USCN Life Science (Catalog no: E94912Hu for SIRT1 and E908868Hu for eNOS, Wuhan/CHINA). Briefly, standards and samples were incubated with antibody coated 96-well plates for 2 hours. After incubation, the substrate solution was added and the plates were incubated for 15–25 min. Then, the reaction was stopped by stop solution. At 450 nm, the intensity of the color change in each well was measured in a microplate reader (Chromate Manager 4300, Palm City/USA).

### Measurement of PON-1 level

The PON-1 level was measured by a fully automated method using a commercially available kit (Rel Assay Diagnostics, Gaziantep/TURKEY). After mixing the sample with appropriate amount of reagents, linear increase of the absorbance of p-nitrophenol which is produced from paraoxon was recorded at 412 nm and nonenzymatic hydrolysis of paraoxon was subtracted from the total rate of hydrolysis. The PON-1 level was calculated in terms of U/L (Rel Assay Diagnostics, Gaziantep/TURKEY).

### Measurement of total antioxidant and oxidant status and determination of oxidative stress index

Total antioxidant status (TAS) and total oxidant status (TOS) of serum were determined using an automated measurement method by an automated analyzer (Chromate Manager 4300, Palm City/USA) as described earlier [25, 26]. In the measurement of TAS, absorbance of the colored dianisidyl radicals was monitored to determine the rates of Fenton reaction which initiates free-radical reactions with the production of a hydroxyl radical. Then, the antioxidative effect of sample against potent free-radical reactions was measured in terms of mmol equiv/L Trolox (Rel Assay Diagnostics, Gaziantep/TURKEY).

In the measurement of TOS, intensity of the colored complex produced by the reaction of ferric ions, which were oxidized from ferrous iono-dianisidine complex due to presence of oxidants, with xylenol orange in an acidic medium was used to determine the total amount of oxidant molecules in the sample. The calibration of assay is performed with hydrogen peroxide. TOS values are expressed in terms of micromolar hydrogen peroxide (H_2_O_2_) equivalents per liter (μmol H_2_O_2_ equiv/L) (Rel Assay Diagnostics, Gaziantep/TURKEY).

Oxidative stress index (OSI) was calculated by the following formula [15, 27]:

OSI=[(TOS)/(TAS×1000)]×100

### DNA isolation and determination of *SIRT1* gene polymorphisms

The genomic DNA was isolated from peripheral blood leukocytes of blood samples taken from all subjects using DNA isolation kit (Invitrogen, Carlsbad, USA). All purified DNA samples were stored at 4°C until PCR application were performed [28, 29].

As described previously, the studied *SIRT1* SNPs (rs7895833 A>G in the promoter region, rs7069102 C>G in intron 4 and rs2273773 C>T in exon 5) were analyzed using PCR-CTPP assay with minor modifications [30, 31].

**Fig 1:**
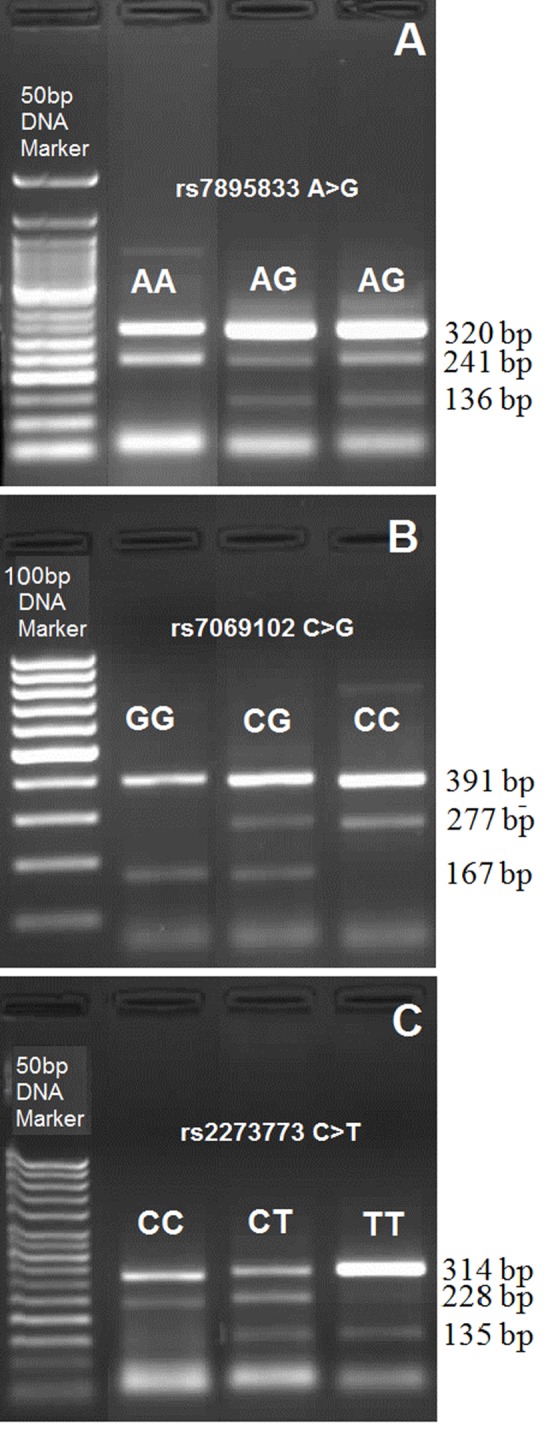
Representative PCR gel pictures of SIRT1 gene polymorphisms. **A)** The genotype for rs7895833 SNP of *SIRT1* gene. **B)** The genotype for rs7069102 SNP of *SIRT1* gene. **C)** The genotype for rs2273773 SNP of *SIRT1* gene. The first lanes of each gel contain a 50 bp DNA ladder.

The *SIRT1* gene segments encompassing rs7895833 A>G, rs7069102 C>G, rs2273773 C>T polymorphic sites were amplified by PCR using the appropriate primers (Table 2) as described previously [2, 15]. Briefly, 25 μl total PCR mixtures (100–200 ng DNA, 10.0 pmol of each primers, 1.0 mM deoxynucleotide triphosphates (dNTPs), 25 mM MgCl_2_ and 2.5U Taq DNA polymerase) in the supplied reaction buffer (Taq Buffer with (NH_4_)_2_SO_4_) were prepared. PCR was performed with the initial denature at 95°C for 10 min.; 30 cycles of 95°C for 1 min., 64°C for **rs7895833 A>G polymorphism**, 62°C for **rs7069102 C>G polymorphism,** 63°C for **rs2273773 C>T polymorphism** for 1 min., and 72°C for 1 min. and additionally the final step at 72°C for 5 min. A 2% agarose gel with ethidium bromide staining was used for visualization of PCR products. Three genotypes for each polymorphism were defined by 3 distinct banding patterns; for **rs7895833 A>G polymorphism:** 320, 241 bp for AA genotype; 320, 241 and 136 bp for AG genotype; and 320, 136 bp for GG genotype (Fig. 1A); for **rs7069102 C>G polymorphism:** 391, 277 bp for CC genotype; 391, 277, 167 bp for CG genotype; and 391, 167 bp for GG genotype (Fig. 1B); for **rs2273773 C>T polymorphism:** 314, 228 bp for CC genotype; 314, 228, 135 bp for CT genotype; and 314, 135 bp for TT genotype (Fig. 1C) [2].

**Table 2:** Primer sequences in *SIRT1* gene polymorphisms.

**Polymorphism**	**Primer Sequences**
rs7895833 A>G	Forward primer 1: CCCAGGGTTCAACAAATCTATGTTG
	Forward primer 2: GGTGGTAAAAGGCCTACAGGAAA
	Reverse primer 1: GCTTCCTAATCTCCATTACGTTGAC
	Reverse primer 2: CCTCCCAGTCAACGACTTTATC
rs7069102 C>G	Forward primer 1: GTAGCAGGAACTACAGGCCTG
	Forward primer 2: GAGAAGAAAGAAAGGCATAATCTCTGC
	Reverse primer 1: CTATCTGCAGAAATAATGGCTTTTCTC
	Reverse primer 2: GATCGAGACCATCCTGGCTAAG
rs2273773 C>T	Forward primer 1: GTGTGTCGCATCCATCTAGATAC
	Forward primer 2: CTCTCTGTCACAAATTCATAGCCT
	Reverse primer 1: GTAGTTTTCCTTCCTTATCTGACAG
	Reverse primer 2: CTGAAGTTTACTAACCATGACACTG

### Statistical evaluation

The results were presented as means ± SEM. Statistical analyses of differences in the distribution of the genotypes or alleles in *SIRT1* gene between age groups were tested by Chi-Square (χ2) test using a standard software package (SPSS 18 for Windows; SPSS Inc., Chicago, IL, USA). Levels of proteins, TAS, TOS and OSI and the relation between mutagenicity and protein levels were compared by One way ANOVA following with Post Hoc Tukey’s test and Pearson’s correlation test using the same software. Receiver-operating characteristics (ROC) analysis by calculating z-test for comparison ROC curves was used to show the strength of the analyzed proteins’ expressions to differentiate distinct age groups. A p value of less than 0.05 was regarded as being statistically significant.

## Results

### Expression levels of SIRT1, eNOS and PON-1 protein and levels of TAS, TOS, OSI, cholesterol and their correlation with age

**Fig 2:**
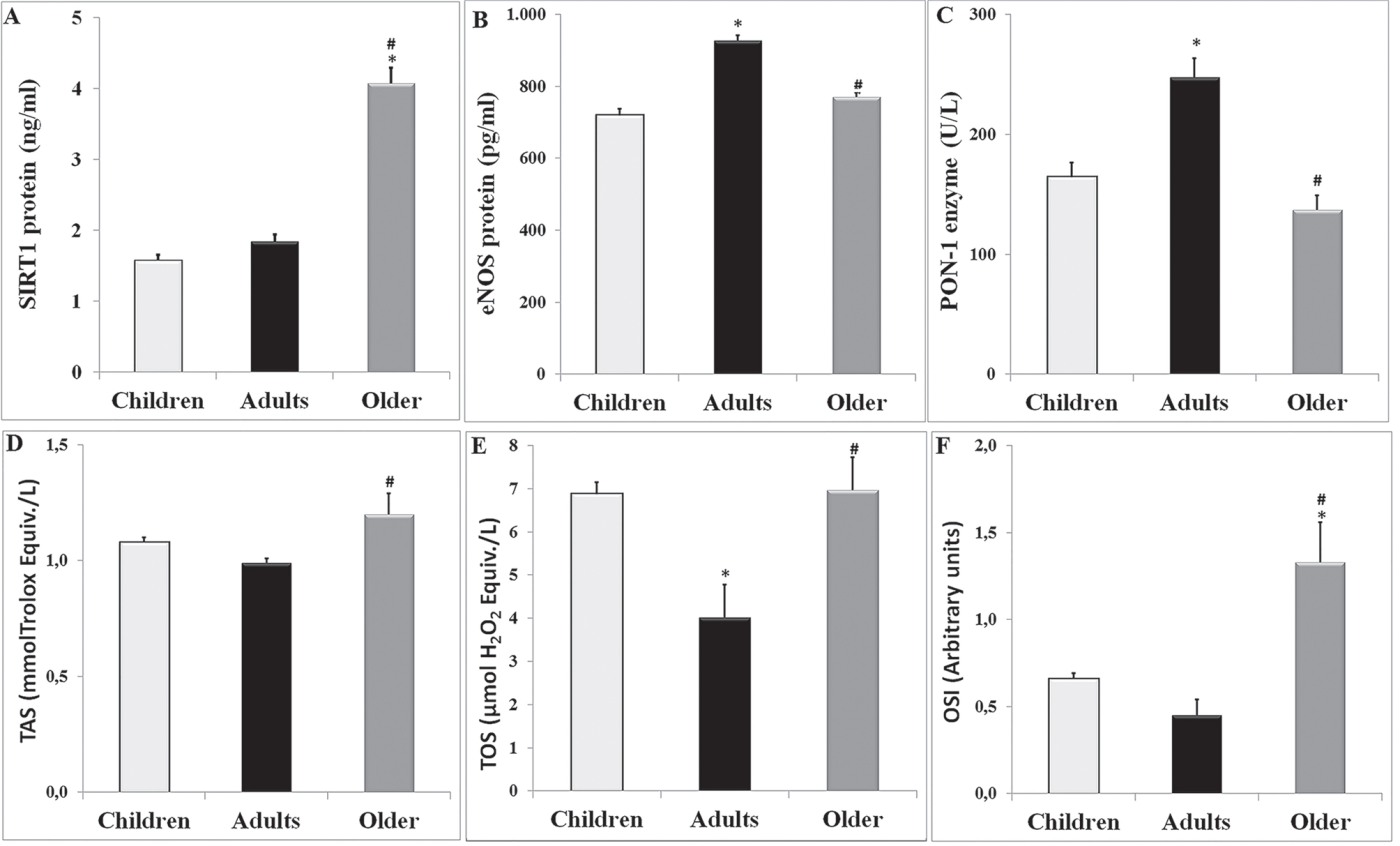
Levels of SIRT1, eNOS, PON-1, TAS, TOS, and OSI in children, adults, and older people. The results are shown as mean ± Standart error of mean (SEM). * indicates significant difference against to children, # indicates significant difference against to adults. *p<0.05 and #p<0.05. Statistical evaluation by One-way ANOVA with post hoc Tukey’s test.

**Fig 3:**
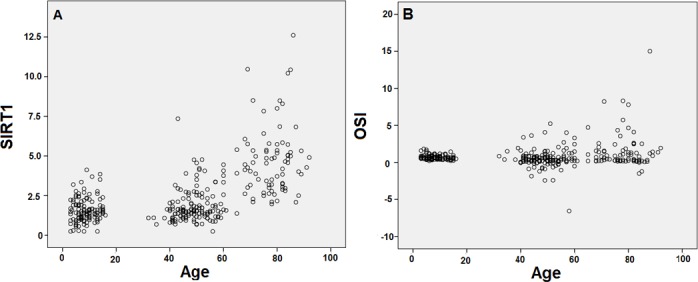
Scatter plot figures for Pearson’s correlation of age with expression levels of SIRT1 and OSI level in overall population. SIRT1 level is expressed as ng/ml and OSI is shown in arbitrary units.

SIRT1 protein expression level and its correlation with age, levels of eNOS and PON-1 proteins, TAS, TOS and OSI were demonstrated in Fig. 2A and Table 3. Also, the correlation between examined proteins and cholesterol levels were presented in Table 3. The level of SIRT1 protein was significantly higher in older people (4.07 ± 0.22 ng/ml) compared with both children (1.58 ± 0.07 ng/ml) and adults (1.84 ± 0.10 ng/ml) (p<0.001). Pearson’s correlation test showed that there was a significant positive correlation between SIRT1 level with age in both adults (p = 0.049) and older people (p<0.001). Also, there were a negative correlation between SIRT1 level with LDL cholesterol in adults (p = 0.014) and a positive correlation between SIRT1 level with HDL cholesterol in older people (p = 0.029). Moreover, Pearson’s correlation test done to the all studied population showed that there was a significant positive correlation between age and SIRT1 (r: 0.573, p<0.001) and OSI (r: 0.169, p = 0.002) shown in a scatter plot (Fig. 3).

**Table 3:** The results of Pearson's correlation of SIRT1, eNOS and PON-1 with age, TAS, TOS and OSI levels.

	**Children (n = 120)**		**Adults (n = 115)**		**Older (n = 103)**	
**SIRT1 correlations with**	**r**	***p***	**r**	***p***	**r**	***p***
Age	0.114	0.217	0.184*	0.049	0.481*	<0.001
eNOS protein (pg/ml)	0.302*	0.001	−0.021	0.826	−0.187	0.059
PON-1protein (U/L)	0.061	0.505	0.039	0.676	−0224*	0.023
TAS (mmolTrolox Equiv./L)	−0.004	0.962	0.109	0.247	−0.029	0.774
TOS (μmol H_2_O_2_ Equiv./L)	−0.173	0.059	0.030	0.749	−0.054	0.589
OSI	−0.129	0.159	0.014	0.882	0.127	0.202
Total cholesterol (mg/dL)	−0,086	0.348	−0.215	0.137	−0.079	0.455
HDL cholesterol (mg/dL)	0.031	0.737	0.277	0.079	0.227*	0.029
LDL cholesterol (mg/dL)	−0.133	0.148	−0.243*	0.014	−0.178	0.074
**eNOS correlations with**	**r**	**p**	**r**	**p**	**r**	**p**
Age	0.053	0.563	0.005	0.958	−0.339*	<0.001
SIRT1 protein (ng/ml)	0.302*	0.001	−0.021	0.826	−0.187	0.059
PON-1protein (U/L)	0.051	0.580	0.149	0.112	0.068	0.496
TAS (mmolTrolox Equiv./L)	0.154	0.094	0.095	0.310	−0.045	0.651
TOS (μmol H2O2 Equiv./L)	−0.002	0.980	0.073	0.436	−0.159	0.109
OSI	−0.050	0.590	0.050	0.598	−0.155	0.119
Total cholesterol (mg/dL)	0.120	0.191	−0.124	0.396	0.145	0.169
HDL cholesterol (mg/dL)	0.203*	0.026	0.195	0.223	0.088	0.400
LDL cholesterol (mg/dL)	0.042	0.647	0.027	0.790	0.236*	0.018
**PON-1 correlations with**	**r**	**p**	**r**	**p**	**r**	**p**
Age	0.117	0.214	−0.016	0.866	−0273*	0.005
SIRT1 protein (ng/ml)	0.061	0.505	0.039	0.676	−0224*	0.023
eNOS protein (pg/ml)	0.051	0.580	0.149	0.112	0.068	0.496
TAS (mmolTrolox Equiv./L)	0.089	0.333	0.096	0.305	−0.080	0.421
TOS (μmol H2O2 Equiv./L)	0.060	0.515	0.040	0.674	0.004	0.965
OSI	0.024	0.791	0.005	0.958	−0.037	0.711
Total cholesterol (mg/dL)	0.324*	<0.001	0.085	0.561	−0.013	0.905
HDL cholesterol (mg/dL)	0.447*	<0.001	−0.245	0.123	0.185	0.075
LDL cholesterol (mg/dL)	0.157	0.086	−0.005	0.959	0.043	0.669

n, number of individuals. Statistical evaluation by Pearson’s Correlation. The results are shown as mean ± SEM. HDL, high density lipoprotein; LDL, low density lipoprotein * p<0.05.

eNOS protein expression level and its correlation with age, levels of SIRT1 and PON-1 proteins, TAS, TOS, and OSI were presented in Fig. 2B and Table 3. The level of eNOS protein was significantly higher in adults (926.61 ± 14.52 pg/ml) compared with both children (719.92 ± 18.04 pg/ml) and older people (770.82 ± 12.21 pg/ml) (p<0.001). However, there was no significant difference in eNOS expression between children and older people. Pearson’s correlation test showed a significant negative correlation between eNOS level and age (p<0.001) and a significant positive correlation between eNOS level and LDL cholesterol (p = 0.018) in older people. Also, there was a positive correlation between eNOS level and HDL cholesterol (p = 0.026) in children. Furthermore, a significant positive correlation between SIRT1 and eNOS level was only observed in children (p = 0.001).

The PON-1 level and its relation with age, levels of SIRT1 and eNOS proteins, TAS, TOS, and OSI were presented in Fig. 2C and Table 3. The level of PON-1 was significantly higher in adults (247.63 ± 15.54 pg/ml) compared with both children (164.49 ± 11.74 pg/ml) and older people (136.90 ± 12.03 pg/ml) (p<0.001). However, there was no significant difference in the PON-1 expression between children and older people. Pearson’s correlation test showed a significant negative correlation between PON-1 level and both age (p = 0.005) and SIRT1 level (p = 0.023) in older people and a significant positive correlation between PON-1 level and both total cholesterol level (p<0.001) and HDL cholesterol level (p<0.001).

The levels of oxidative stress parameters, TAS, TOS, and OSI, were presented in Fig. 2D, 2E, and 2F. In older people (TAS: 1.20 ± 0.09, TOS: 6.96 ± 0.77, and OSI: 1.33 ± 0.23), these three parameters were significantly higher than those of adults (TAS: 0.99 ± 0.02, TOS: 4.01 ± 0.76, and OSI: 0.45 ± 0.09) (p = 0.017, p = 0.001, and p<0.001, respectively). Furthermore, OSI levels of older people (1.33 ± 0.23) also significantly increased compared with OSI levels of children (0.66 ± 0.03). Interestingly, TOS levels of adults (4.01 ± 0.76) were significantly lower than TOS levels of both children (6.88 ± 0.27) and older people (6.96 ± 0.77) (p = 0.001). In aspect of TAS level between older people (1.20 ± 0.09) and children (1.08 ± 0.02), there was no significant difference.

### Frequencies of *SIRT1* (rs7895833 A>G, rs7069102 C>G, rs2273773 C>T) gene polymorphisms

The frequencies of genotypes and alleles in *SIRT1* gene in all groups are shown in Table 4. For rs7895833 A>G in promoter region, there was no individual carrying GG mutant genotype at any age. The frequency of AG genotype was significantly higher in older people compared with adults and children (p = 0.014).

**Table 4:** Distribution of rs7895833 A>G, rs7069102 C>G and rs2273773 C>T genotypes and alleles in study groups.

	**Children (n = 120)**		**Adults (n = 115)**		**Older (n = 103)**		***Statistical results***	
	**(%)**	**(n)**	**(%)**	**(n)**	**(%)**	**(n)**	**χ^2^**	***p value***
**rs7895833 A>G Genotype**								
AA	66.7	**80**	73.0	**84**	54.4	**56**		
AG	33.3	**40**	27.0	**31**	45.6*	**47**	8.543	0.014
GG	0.0	**0**	0.0	**0**	0.0	**0**		
**Allele**								
A	83.3	**200**	84.7	**199**	79.1	**159**	1.292	0.256
G	16.7	**40**	15.3	**36**	20.9	**42**		
**rs7069102 C>G Genotype**								
CC	4.2	**5**	17.4	**20**	8.7	**9**		
CG	85.8*	**103**	50.4	**58**	60.2	**62**	37.538	<0.001
GG	10.0*	**12**	32.2	**37**	31.1	**32**		
**Allele**								
C	47.1	**113**	42.6	**98**	38.8	**80**	3.072	0.080
G	52.9	**127**	57.4	**132**	61.2	**126**		
**rs2273773 C>T Genotype**								
CC	0.0	**0**	7.8	**9**	0.0	**0**		
CT	100.0	**120**	77.4*	**89**	98.1	**101**	47.771	<0.001
TT	0.0	**0**	14.8	**17**	1.9	**2**		
**Allele**								
C	50	**120**	46.5	**107**	49.0	**101**	0.042	0.838
T	50	**120**	53.5	**123**	51.0	**105**		

n, number of individuals. Statistical evaluation by the Chi-square test. *p<0.05.

For rs7069102 C>G in intron 4, the frequencies of GG genotype were significantly higher in adults and in older people compared with children (p<0.001). Most of the children were carrying heterozygote (CG) genotype (Table 4). Therefore, the rate of having CG genotype was significantly lower in adults and older people compared with children.

For both rs7895833 A>G and rs7069102 C>G, the rate of having G allele was higher in older people as compared with children and adults, however, it did not reach accepted level of significance (Table 4).

For rs2273773 C>T in exon 5, interestingly, in our study population, all children and most of the older people (except only 2 people with TT genotype) were carrying heterozygote (CT) genotype. However, there were people carrying each genotype in adults. Therefore, a significant difference in the rate of having CT genotype and TT genotype appeared between adults and other two age group (p<0.001).

### Relationship between *SIRT1* gene polymorphisms and levels of SIRT1, eNOS, PON-1, cholesterol, TAS, TOS, and OSI

For rs7895833 A>G, rs7069102 C>G, rs2273773 C>T SNPs, the associations of SIRT1, eNOS and PON-1 protein levels and distributions of genotypes are shown in Table 5.

**Table 5:** Comparison of genotypes of rs7895833 A>G, rs7069102 C>G and rs2273773 C>T SNPs with levels of SIRT1, eNOS, and PON-1 protein.

		SIRT1				eNOS				PON-1			
		Children(n = 120)	Adults (n = 115)	Older (n = 103)	*p*	Children (n = 120)	Adults (n = 115)	Older (n = 103)	*p*	Children (n = 120)	Adults (n = 115)	Older (n = 103)	*p*
**rs7895833**	AA **(n)**	1.56 ± 0.09 **(80)**	1.91 ± 0.12 **(84)**	3.62 ± 0.30*^#^ **(56)**	**<0.001**	742.1 ± 19.3 **(80)**	923.3 ± 18.8* **(84)**	782.1 ± 16.0^#^ **(56)**	**<0.001**	165.3 ± 15.3 **(80)**	256.3 ± 18.5* **(84)**	153.9 ± 18.0^#^ **(56)**	**<0.001**
	AG **(n)**	1.61 ± 0.12 **(40)**	1.64 ± 0.15 **(31)**	4.61 ± 0.32*^#^ **(47)**	**<0.001**	675.6 ± 37.3 **(40)**	935.6 ± 17.9* **(31)**	757.4 ± 18.8^#^ **(47)**	**<0.001**	162.8 ± 17.9 **(40)**	224.3 ± 28.7 **(31)**	116.7 ± 14.9^#^ **(47)**	**0.001**
	GG **(n)**	0.0 ± 0.0 **(0)**	0.0 ± 0.0 **(0)**	0.0 ± 0.0	-	0.0 ± 0.0	0.0 ± 0.0	0.0 ± 0.0	-	0.0 ± 0.0	0.0 ± 0.0	0.0 ± 0.0	-
				**(0)**		**(0)**	**(0)**	**(0)**		**(0)**	**(0)**	**(0)**	
**rs7069102**	CC **(n)**	1.40 ± 0.34 **(5)**	1.65 ± 0.19 **(20)**	3.37 ± 0.60*^#^ **(9)**	**0.002**	563.3 ± 138.1 **(5)**	938.9 ± 21.5* **(20)**	749.9 ± 34.8^#^ **(9)**	**<0.001**	126.2 ± 60.2 **(5)**	245.5 ± 38.4 **(20)**	214.9 ± 56.6 **(9)**	**0.370**
	CG **(n)**	1.58 ± 0.08 **(103)**	1.82 ± 0.13 **(58)**	4.27 ± 0.27*^#^ **(62)**	**<0.001**	725.1 ± 19.2 **(103)**	919.5 ± 17.0* **(58)**	757.5 ± 13.3^#^ **(62)**	**<0.001**	160.5 ± 12.3 **(103)**	256.9 ± 21.4* **(58)**	128.4 ± 15.5^#^ **(62)**	<0.001
	GG **(n)**	1.66 ± 0.15 **(12)**	1.95 ± 0.21 **(37)**	3.88 ± 0.47*^#^ **(32)**	**<0.001**	740.8 ± 46.4 **(12)**	931.1 ± 35.0* **(37)**	802.4 ± 27.7^#^ **(32)**	**0.002**	214.6 ± 46.2 **(12)**	234.3 ± 28.6 **(37)**	101.5 ± 17.9^#^ **(32)**	**0.016**
**rs2273773**	CC **(n)**	0.0 ± 0.0 **(0)**	1.83 ± 0.36 **(9)**	0.0 ± 0.0	-	0.0 ± 0.0	941.2 ± 18.9 **(9)**	0.0 ± 0.0	-	0.0 ± 0.0	200.4 ± 55.7 **(9)**	0.0 ± 0.0	-
				**(0)**		**(0)**		**(0)**		**(0)**		**(0)**	
	CT	1.58 ± 0.07 **(120)**	1.75 ± 0.10 **(89)**	4.13 ± 0.22*^#^ **(101)**	**<0.001**	719.9 ± 18.0 **(120)**	918.4 ± 18.0* **(89)**	775.1 ± 10.7*^#^ **(101)**	**<0.001**	164.5 ± 11.7 **(120)**	246.6 ± 17.7* **(89)**	138.3 ± 12.2^#^ **(101)**	**<0.001**
	**(n)**												
	TT **(n)**	0.0 ± 0.0 **(0)**	2.31 ± 0.37 **(17)**	0.85 ± 0.60	-	0.0 ± 0.0	962.1 ± 25.2 **(17)**	555.7 ± 399.9 **(2)**	-	0.0 ± 0.0	278.0 ± 41.2 **(17)**	66.0 ± 38.0	-
				**(2)**		**(0)**				**(0)**		**(2)**	

n, number of individuals. Statistical evaluation by One Way ANOVA following Tukey’s test. *indicates significant difference against to children, ^#^ indicates significant difference against to adults. * p<0.05 and ^#^p<0.05.

For rs7895833 A>G, older people carrying both wild-type (AA) genotype and heterozygote (AG) genotype had significantly higher SIRT1 expression level compared with both children and adults (p<0.001). On the contrary, adults carrying both wild-type (AA) genotype and heterozygote (AG) genotype had significantly higher eNOS expression level compared with both children and older people (p<0.001). Interestingly, older people carrying heterozygote mutant (AG) genotype had significantly higher SIRT1 level compared with older people carrying wild-type (AA) genotype (p = 0.026). Parallel to this, the average age of older people carrying AG genotype (76.0 ± 1.5 years) was significantly higher than the average age of older people carrying AA genotype (71.3 ± 1.4 years) (p = 0.021).

For rs7069102 C>G, older people carrying all type of genotypes had significantly higher SIRT1 level than those of adults and children. Similarly, adults carrying all type of genotypes had significantly higher eNOS level than those of older people and children (p<0.001).

For rs2273773 C>T, only SIRT1 and eNOS levels for people carrying heterozygote genotype (CT) could be analyzed statistically due to lack of people carrying homozygote wild-type and mutant (CC and TT) genotypes in our population. Correspondingly, older people had significantly higher SIRT1 level than children and adults, and adults had significantly higher level for eNOS than children and older people.

PON-1 level was significantly higher in adults carrying wild-type (AA) genotype for rs7895833, heterozygote (CG) genotype for rs7069102, heterozygote (CT) genotype for rs2273773 than both children and older people (p<0.001). However, older people carrying AG genotype for rs7895833 and GG genotype for rs7069102 had significantly lower level of PON-1 compared with adults (p = 0.001 and p = 0.016, respectively).

In addition, older people carrying GG genotype (119.2 ± 6.8 mg/dl) had significantly higher level of LDL cholesterol than those of older people carrying CG genotype (99.4 ± 4.5 mg/dl) for rs7069102, whilst there was no significant association between other genotypes of studied SNPs and cholesterol levels.

For rs7895833 A>G, rs7069102 C>G, rs2273773 C>T SNPs, the associations of TAS, TOS and OSI levels and distributions of genotypes are shown in Table 6. The TAS level was significantly higher in older people carrying wild-type (AA) genotype for rs7895833 and carrying heterozygote (CG) genotype for rs7069102 as compared with younger people. The TOS level was significantly lower in adults carrying heterozygote genotypes for all three SNPs and in adults carrying wild-type (AA and CC) genotypes for both rs7895833 and rs7069102 compared with children and older people. In older people carrying all type of genotypes, OSI levels were significantly higher than younger people. However, there was no significant difference in the OSI level in people carrying wild type genotype for rs7895833.

**Table 6:** Comparison of genotypes of rs7895833 A>G, rs7069102 C>G and rs2273773 C>T SNPs with levels of TAS, TOS and OSI.

		TAS				TOS				OSI			
		Children(n = 120)	Adults (n = 115)	Older (n = 103)	*p*	Children (n = 120)	Adults (n = 115)	Older (n = 103)	*p*	Children (n = 120)	Adults (n = 115)	Older (n = 103)	*p*
**rs7895833**	AA **(n)**	1.08 ± 0.02 **(80)**	1.00 ± 0.03 **(84)**	1.36 ± 0.12 ^*^^#^ **(56)**	**<0.001**	7.05 ± 0.34 **(80)**	4.29 ± 0.95^*^ **(84)**	6.76 ± 1.25 ^*^ **(56)**	**<0.037**	0.67 ± 0.03 **(80)**	0.49 ± 0.22 **(84)**	0.84 ± 0.22 **(56)**	**0.172**
	AG **(n)**	1.09 ± 0.03 **(40)**	0.96 ± 0.04 **(31)**	1.01 ± 0.13 **(47)**	**<0.662**	6.56 ± 0.40 **(40)**	3.25 ± 1.14^*^ **(31)**	7.20 ± 0.83^#^ **(47)**	**<0.003**	0.64 ± 0.05 **(40)**	0.33 ± 0.13 **(31)**	1.91 ± 0.41^*^^#^ **(47)**	**<0.001**
	GG **(n)**	0.0 ± 0.0 **(0)**	0.0 ± 0.0 **(0)**	0.0 ± 0.0	-	0.0 ± 0.0 **(0)**	0.0 ± 0.0	0.0 ± 0.0	-	0.0 ± 0.0 **(0)**	0.0 ± 0.0 **(0)**	0.0 ± 0.0	-
				**(0)**			**(0)**	**(0)**				**(0)**	
**rs7069102**	CC **(n)**	1.02 ± 0.08 **(5)**	0.97 ± 0.06 **(20)**	0.96 ± 0.25	**0.970**	7.54 ± 0.67 **(5)**	2.21 ± 1.08 **(20)**	6.73 ± 1.75^#^ **(9)**	**0.020**	0.76 ± 0.10 **(5)**	0.24 ± 0.11 **(20)**	1.00 ± 0.34^#^ **(9)**	**0.015**
				**(9)**									
	CG **(n)**	1.03 ± 0.02 **(103)**	1.00 ± 0.04 **(58)**	1.35 ± 0.13^*^^#^ **(62)**	**0.001**	6.95 ± 0.30 **(103)**	4.49 ± 1.01^*^ **(58)**	7.94 ± 0.90^#^ **(62)**	**0.004**	0.66 ± 0.03 **(103)**	0.47 ± 0.11 **(58)**	1.33 ± 0.28^#^ **(62)**	**0.001**
	GG **(n)**	1.07 ± 0.07 **(12)**	1.07 ± 0.03 **(37)**	0.97 ± 0.14 **(32)**	**0.842**	6.00 ± 0.46 **(12)**	4.22 ± 1.66 **(37)**	5.14 ± 1.70 **(32)**	**0.821**	0.60 ± 0.06 **(12)**	0.52 ± 0.23 **(37)**	1.43 ± 0.49^*^^#^ **(32)**	**0.149**
**rs2273773**	CC **(n)**	0.0 ± 0.0 **(0)**	1.05 ± 0.08 **(9)**	0.0 ± 0.0	-	0.0 ± 0.0 **(0)**	3.88 ± 4.60 **(9)**	0.0 ± 0.0	-	0.0 ± 0.0 **(0)**	0.36 ± 0.54 **(9)**	0.0 ± 0.0	-
				0.0 ± 0.0 **(0)**	0.0 ± 0.0 **(0)**			0.0 ± 0.0 **(0)**					
	CT	1.08 ± 0.02 **(120)**	0.97 ± 0.03 **(89)**	1.20 ± 0.09^#^ **(101)**	**0.023**	6.88 ± 0.27 **(120)**	4.08 ± 0.84^*^ **(89)**	6.99 ± 0.79^#^ **(101)**	**0.002**	0.66 ± 0.03 **(120)**	0.47 ± 0.11 **(89)**	1.35 ± 0.23^*^^#^ **(101)**	**<0.001**
	**(n)**												
	TT **(n)**	0.0 ± 0.0 **(0)**	1.02 ± 0.06 **(17)**	1.17 ± 0.17	-	0.0 ± 0.0 **(0)**	3.71 ± 1.35 **(17)**	5.45 ± 0.45 **(2)**	-	0.0 ± 0.0 **(0)**	0.36 ± 0.15 **(17)**	0.47 ± 0.03 **(2)**	-
				**(2)**								**(2)**	

n, number of individuals. Statistical evaluation done by the Student’s t test and One Way ANOVA following Tukey’s test. *indicates significant difference against to children, ^#^ indicates significant difference against to adults. * p<0.05 and ^#^p<0.05.

### Receiver operating characteristic (ROC) curves of SIRT1, eNOS and PON-1 protein to differentiate distinct age groups

The levels of SIRT1, eNOS and PON-1 protein in children, adults and older people were analyzed by ROC analysis to show the strength of the analyzed protein expressions to differentiate distinct age groups. For comparing adults with children, the ROC curves suggest that eNOS (area under the curve (AUC): 0.857 [95% CI 0.806–0.899]) had better sensitivity and specificity than either PON-1 (AUC: 0.661 [95% CI 0.596–0.721]) or SIRT1 (AUC: 0.558 [95% CI 0.492–0.623]). For comparing older people with children, the ROC curves suggest that SIRT1 (AUC: 0.879 [95% CI 0.829–0.919]) had better sensitivity and specificity than either PON-1 (AUC: 0.577 [95% CI 0.509–0.642]) or eNOS (AUC: 0.547 [95% CI 0.479–0.613]). For comparing adults with older people, the ROC curves suggest that eNOS (AUC: 0.855 [95% CI 0.801–0.899]) had better sensitivity and specificity than either PON-1 (AUC: 0.718 [95% CI 0.653–0.777]) or SIRT1 (AUC: 0.843 [95% CI 0.788–0.889]).

**Fig 4:**
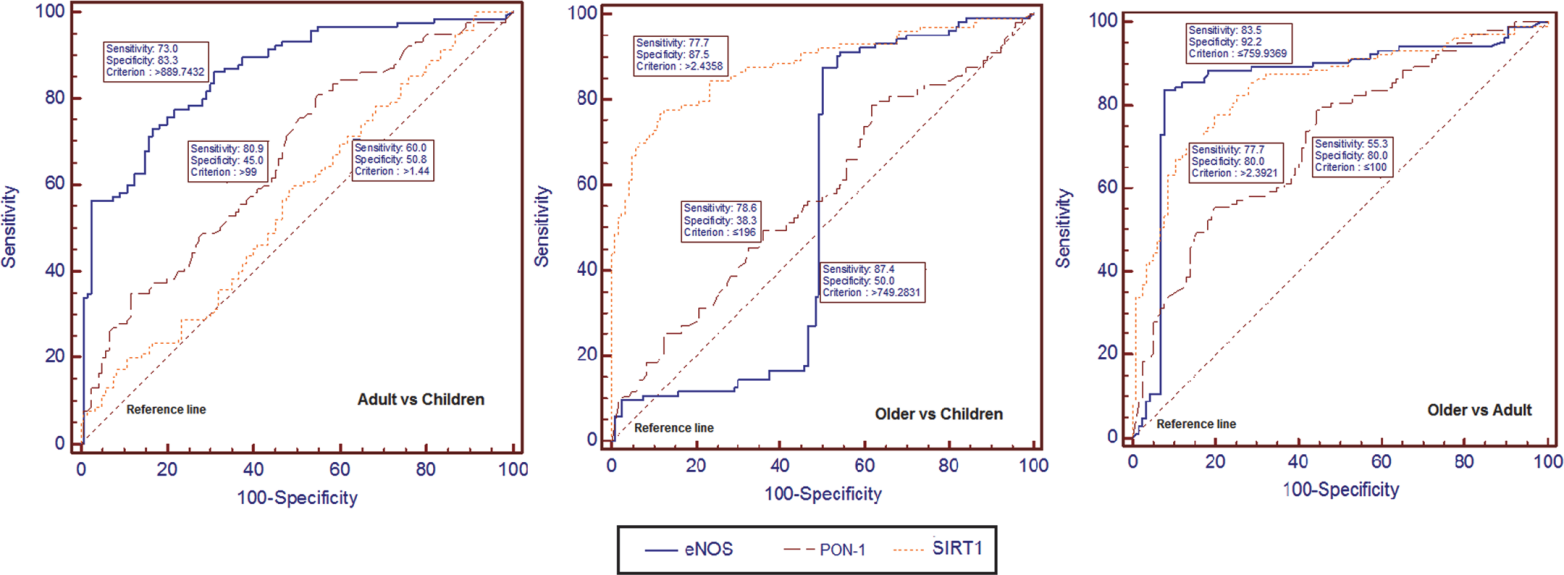
Receiver-operating characteristic (ROC) curves for protein expressions. ROC curves differentiate distinct age groups (Adult vs Children, Older vs Children, and Older vs Adult) for the strength of the expressions of SIRT1, eNOS, and PON-1.

An eNOS level>889.743 pg/mL was highly sensitive and specific for differentiating adults from children (sensitivity 73.0%, specificity 83.3%) and eNOS level<759.937 pg/mL was highly sensitive and specific for differentiating adults from older people (sensitivity 83.5%, specificity 92.2%) (Fig. 4). A SIRT1 value>2.4358 ng/mL as the cut-off value, differentiated older people from children (sensitivity 77.7%, specificity 87.5%).

## Discussion

Aging is defined as the accumulation of progressive organ dysfunction. Therefore, controlling the rate of aging by clarification the complex pathways has a significant clinical importance. In the present study, we investigated how SIRT1 protein level, the product of longevity gene, changes with aging in response to the finding of one of our studies presenting a positive correlation with SIRT1 protein and age [15]. In the present study, we analyzed the *SIRT1* gene polymorphisms which were studied by us previously, and its relation with levels of SIRT1, eNOS, PON-1, cholesterol, TAS, TOS, and OSI to demonstrate the association between genetic variation and phenotype at different ages in humans. Therefore, the current study is important for a much better molecular understanding of the role of longevity gene *SIRT1* and its protein product in aging in humans and, due to importance of epigenetics in several age related diseases, developing essential life coaching strategies to provide a healthy aging for humans.

Previous studies done in animals showed an association between sirtuins and lifespan elongation [16, 18]. Most of these studies done by calorie intake restriction as an intervention have explained life span elongation by inducement of a defensive response such as increasing cell defense to toxins and free radicals for slowing down apoptosis or increasing cell repair which are important factors in aging [32–34]. Therefore, increased SIRT1 expression and activity may be effective for keeping the cells and organs functioning properly for longer times. In the current study, we observed a significant increase in SIRT1 protein level in older people (above 55 years) compared with younger people (children and adults). With a cut-off point of ≤2.44 and ≤2.39 sensitivity and specificity of the level of SIRT1 were higher to differentiate older people from children and adult, respectively (Fig. 4). Also, there was a significant positive correlation between SIRT1 level and age in older people.

According to our present results, absence of significant change in SIRT1 level between children and adults (from age 3 to 55) may suggest that SIRT1 levels are controlled during a long period of times in our lives and its expression dramatically increases in older ages. Previous studies done in animals showed that aging increases the amount of SIRT1 protein level besides a decline in the SIRT1 activity in mice which could be related with oxidative damage [35, 36]. Ramsey et al. [37] suggested that reduction in sirtuin activity in mice may be related with a decline in NAD^+^ levels with aging. During oxidative stress, the NAD^+^-dependent DNA repair enzyme, poly(ADP-ribose) polymerase-1 (PARP), is activated and decreases NAD^+^ level which increases aging [38]. Also, in the current study, OSI level was increased in older people suggesting an increase in oxidative stress. Therefore, increased protein level of SIRT1 in older people may be a compensatory mechanism to compete the age- and oxidative stress-related decrease in NAD^+^ levels. Also, the positive relation between SIRT1 and HDL cholesterol levels endorsed this compensatory mechanism against to increased oxidative stress in elderly [39, 40].

eNOS maintains vascular integrity during aging by activating vasorelaxation and angiogenesis [41]. SIRT1/eNOS axis is a potential target for vascular senescence [42]. It was also stated that vascular aging is accompanied by reduced eNOS expression/activity [43]. In the present study, we observed decreased protein level of eNOS in older people compared with adults. However, there was no significant difference in eNOS level between older people and children. With a cut-off point of >899.74 and >759.94 sensitivity and specificity of the level of eNOS were higher to differentiate adults from children and older people, respectively (Fig. 4).

In literature, there are controversial claims for the change in eNOS expression level during aging. In both animal and human studies, some researchers found either increase/decrease [44–47] or insignificant changes in the eNOS expression during aging [48, 49]. It was previously reported that SIRT1 colocalizes with eNOS in endothelial cells and deacetylates eNOS directly [50]. Indeed, it was observed that co-overexpression of SIRT1 and eNOS significantly inhibited the senescence-like phenotype [51]. In the current study, we observed a positive correlation between SIRT1 and eNOS only in children. In older people we found a negative correlation between the expression levels of SIRT1 and eNOS (p = 0.059).

Demonstration of genetic variations gives a hint to people for changing their lifestyles to decrease the age-related diseases and increase their lifespan. In spite of several *SIRT1* polymorphism studies on BMI, obesity, diabetes and cardiovascular diseases [2, 52–60], there is a few number of *SIRT1* SNPs studies on life span longevity in humans [56, 61–64]. Therefore, research on the molecular relation between *SIRT1* gene and aging is immediately needed to prevent age-related diseases by life coaching related with modifiable risk factors like eating habits and physical activity. In the present study, we found a significant increase in the frequency of heterozygote (AG) genotype in older people for rs7895833 among the studied SNPs. Parallel to this, the SIRT1 level of older people carrying heterozygote AG genotype for rs7895833 was the highest compared with both children and adults. Also, the average age for this genotype was the highest compared with both other genotypes of this SNP and all genotypes of other SNPs. Therefore, this result may point out the strong relation of rs7895833 with aging. Further investigation is necessary to verify its role in life span elongation. Furthermore, for rs7069102 C>G, a significant increase in the frequencies of GG genotype was observed in adults and in older people compared with children who were mostly carrying heterozygote (CG) genotype (Table 4). In other words, according to present results, the rate of carrying mutant genotype (GG) increases with age in our study population whereas Figarska et al. [56] found no significant associations between genotypes and mortality risk for rs7069102 in Dutch people.

The “free-radical theory of aging” is known as the most famous aging mechanism which suggesting oxidative stress related damage with advanced aging due to an imbalance between the concentrations of free radicals and antioxidants [65]. Therefore, we measured the levels of TAS, TOS and OSI to investigate the relation between these oxidative stress parameters with *SIRT1* gene polymorphism during aging. In this study, OSI level in older people was significantly higher compared with adults and children. During senescence, an elevation in reactive oxygen species (ROS) and/or deficiency of ROS scavenging enzymes increased oxidative DNA damage which is a major factor associated with age-related diseases [66]. As stated previously, oxidative stress induced NAD^+^ depletion due to the activation of PARP plays a significant role in the aging process [35]. In the same study, they presented that total antioxidant capacity declines with aging in mice. In the present study, TAS level in older people was higher than TAS level of adults; however, it was not significantly different from TAS level in children. This may be related with high levels of SIRT1 protein in older people and SIRT1’s role in induction of antioxidants [67, 68].

Furthermore, older people carrying heterozygote (AG) genotype for rs7895833, homozygote mutant (GG) genotype for rs7069102, and heterozygote (CT) genotype for rs2273773 had significantly higher OSI level than those of adults and children suggesting a relation between carrying mutant allele (G or T) in their genotypes and oxidative stress.

PON-1 is an antioxidant enzyme which prevents atherosclerosis by associating with high-density lipoprotein (HDL) [69]. Low activity of this enzyme reduces eNOS activity which increases the risk of cardiovascular diseases [70, 71]. Therefore, PON-1 activity is found essential for SIRT1 expression [72]. In our study, we observed a significant increase in PON-1 level in adults compared with children. It is also previously stated that PON-1 activity is low in children as compared to adults in Mexican-American people [73]. However, in older people, there was a significant decrease in PON-1 level as compared with adults. This may be related with oxidative stress because there are numerous researches which demonstrate the role of PON-1 on the age- and oxidative stress-related diseases such as diabetes and heart diseases [71, 74, 75]. Interestingly, decrease in the PON-1 level was dramatic in older people carrying mutant allele (G) for both rs7895833 and rs7069102 compared with adults. Also, increase in LDL cholesterol level was significant in older people carrying mutant genoype (GG) for rs7069102. These results are consistent with our OSI levels which older people carrying mutant allele (G) had a significantly higher OSI level than adults. In addition, there is a significant negative correlation between SIRT1 level and PON-1 level in older people again suggesting oxidative-stress related increase in SIRT1 level to compensate the decrease in antioxidant enzymes in elderly.

In conclusion, we observed a significant increase in SIRT1 level in older people. Interestingly, in our study population, the oldest people (76.0 ± 1.5 years) carrying AG genotypes for rs7895833 have the highest SIRT1 level (4.61 ± 0.32) suggesting an association between rs7895833 SNP and lifespan longevity. Also, OSI levels were higher and PON-1 levels were lower in older people than adults and children which may explain high levels of SIRT1 protein as a compensatory mechanism for oxidative stress in older people. As it is known that aging is an active continuation of an organism’s genetically programmed development, the genetic studies in the longevity genes and their association with phenotype including our present results which is the first study showing age-related changes in SIRT1 levels are important for a healthy aging by controlling gene-environment interactions at earlier ages. Therefore, further studies are warranted the relation between genetic and epigenetic mechanisms for SIRT1 to increase the quality of late life by reducing the burden of age-related chronic diseases with changing life styles and eating habits.

### Study Limitations

This study has a strength being the first to demonstrate the changes of SIRT1 level in different ages. However, this is an initial Turkish population based study that needs to be larger sample size to better interpret the results for the *SIRT1* polymorphisms. Another draw-back of the current study is imbalance between the numbers of each gender and the absence of determination of SIRT1 activity and its interaction with eNOS. Therefore, future studies with larger sample size with nearly equal number of each sex by measuring activities of studied proteins in addition to their expressions are needed to potentiate the validity of this study.
